# Supplementary material for: Patterns of Intron Gain and Loss in Fungi
Source: PLoS Biol. 2004 Nov 30;2(12):e422. doi: 10.1371/journal.pbio.0020422 (PMC532390; doi:10.1371/journal.pbio.0020422)
Supplement: Table S1 — Also available at http://genes.mit.edu/NielsenEtAl/. (4.3 MB ZIP). [file pbio.0020422.st001.zip › NielsenEtAl/html/1408.html]

AN0409.1.NCU00046.1.MG10199.1.FG07231.1


```
 CLUSTAL W (1.82) Multiple Sequence Alignments - Introns Inserted


Sequence 1: NCU00046.1	853 aa
Sequence 2: MG10199.1	866 aa
Sequence 3: FG07231.1	843 aa
Sequence 4: AN0409.1	807 aa
Alignment Length: 882 aa
Number Identitical Residues: 341 aa
Alignment Score (without introns) 16845


MG10199.1 	---MDSEDGELFIK0QLATFVRTHEKALANALQFRRQNLPRHGSSQSVSSISTSNIATSP
NCU00046.1	---MDSEDGEFFIK0QLASFVRTHEKALANALQFRRQNTPRHASSQSVSVLSTS-APTAS
FG07231.1 	---MDSEDGELFIK0QLAGFVRTHEKALANALQLRRQ--VRHKPSQSTGSAALL-SQTST
AN0409.1  	MEKLDTTDGQLFIK0NLASFVRTHEKALANALQLKRQPTRNGHTTQSG------------
          	 ..:*: **::*** :** **************::**   .  .:**             

MG10199.1 	TAMERPATSSSASSTLAAALSLGTLSFTSASVKSAKLALTSHHLFYLLSRFEELGIPVGP
NCU00046.1	SLPERPSTSSSPSSTLAAALSLGSLNFTSHNVKSAKLALTAHHLFYLLSRFQELAIPVGP
FG07231.1 	SLPERPSTSASTSSSLAAALSLGSLNFTSHNVKSAKLALTPHHLFYLLSRFEELSINVGP
AN0409.1  	------SSSGSTSSTLAAALSFGALKFTSQSVKPAKLTLTPHHLFYLLSRFEEISIAVGP
          	      ::*.*.**:******:*:*.*** .**.***:**.**********:*:.* ***

MG10199.1 	MKVRLENLHDNSSSANYVSFLSQSQRTKSRESDVGSIRSVSSIRSVMSGMSALWASFGIG
NCU00046.1	MKVRLENLHDNRNSGNYVSFLSQTQRCKSRGSDVGSIHSVSSIRSVMSGMSALWSSFGIG
FG07231.1 	MKVRLENLHDSSSSANYVSFLSNTQRSRSKGSDIDSIHSVSSMRSVMSGMSALWSSFGIG
AN0409.1  	MNVRLENIHTDVSQS-YVSFLNKPQRSKG---DRDSIHSVSSVRSVMSGMSSLWSSFGLV
          	*:*****:* . ... *****.:.** :.   * .**:****:********:**:***: 

MG10199.1 	SGVSAARTERQKAAIQADLKYLYSAFTKIPCLKLAPDWRARLIRGYEEFPFDSAVPLYVF
NCU00046.1	SSISAARSEKQKAAIEADLKYLYSAFTKIPCLRLAPDFRARLIKGYEEFPFDSAVPLYVF
FG07231.1 	ASISAARTERQKAALEADMKYLYSAFTKIPCLKLAPDWRARLIRGYEEFPFDSAVPLYVF
AN0409.1  	SKESSSKSEKAKAALEADIKYLYSAFTKIPCLRLAPDRRARLIRGYEEFPFDTAVPLHSF
          	:  *::::*: ***::**:*************:**** *****:********:****: *

MG10199.1 	KNVQALEVCDMDFRHFFGWDRMADQLRSLTLKRASIDDPADILIDIVLDDMDKRRRRSSK
NCU00046.1	KNVQALEVSDTDFRQFFGWDRLSEQLRSLTLKRASIDDPADVLIDIVLDDMDKRRRRSSK
FG07231.1 	KNLQALEVSSIDFRQFFGWDRLADQLRSLTLRRAGIEDPADILIDIVLDDMDKRRRRVSK
AN0409.1  	KNLSALEIIDIDFRSFYGWDRLSEQLRTLTLKRANIEDPAELLTGIVLDDIDKRRRRSSK
          	**:.***: . *** *:****:::***:***:**.*:***::* .*****:****** **

MG10199.1 	TQASPTTPWGAGASSPRRTPAAEHNESHNSTSAPGSPQPRYSTGDLQIGSLNSEASMGGK
NCU00046.1	AQSSPTGPWAA--PSPRRSPTIPQ-AAQKSNSPPSSPDARTSNTEIPVGSLNSESGPDQF
FG07231.1 	SQSSPTSVWAG-SGSPRRN--VAHPELNRAVSAPSSPGP---HIDMRVGSMTP-----SE
AN0409.1  	HQSSPVLGWSG--TTNPRP--VSRHDASGSLSAPGSPVA-----DTALGSSAS-------
          	 *:**.  *..   :  *     :     : *.*.** .     :  :**  .       

MG10199.1 	DDTSDDSRRPSLVRADSGEPSTSPS-KSSRPRSHSPRRPTSSRNSTS-HVRTS-HKIRRS
NCU00046.1	GAALHNHRRPSVVRVDSDE-SRTPA-KDGRPRSHSPRRPTSSRNGSS-NVRGS-HKIRRS
FG07231.1 	HAIEESSRRQSLCKEDENNDEPQPSHKPSRPRSSSPPRPTSSRTQSH-PIRGSHHRMRRS
AN0409.1  	------PQAVSMIRLASEGAKPRPR-----AGSVSPSRPSASKHSSHRHNRGQSSRIRRT
          	       :  *: :  .   .  *      . * ** **::*:  :    * .  ::**:

MG10199.1 	GSGSSHSSLSDSWHGHHRG-GSTSNLLSVGILPASKWRFLKHLSLADNSLTCISATSLAP
NCU00046.1	GSGSSHSSLSDSWYNSR---GSSSNLLAMGTLPPSKWRFLKHLSLADNSLSSIPAASLAP
FG07231.1 	GSGSSQSSLSDSWTGHHHSRGSSGNLLAMGVLPASKWRFLRHLSLADNSMTCIPRNSLAP
AN0409.1  	GSGSSNSSENSGYR-----TGSSSNLLAT-VLPPSKWRFLRHLGLADNSLTSVSAAGLAP
          	*****:** ...:       **:.***:   **.******:**.*****::.:.  .***

MG10199.1 	LSNTLHSLDLSANLFTQIPDSLATLTALRALNLSHCMIDSLHSLLRNPLPAITALNLRAN
NCU00046.1	LANSLHSLDLSANLFTQIPDSLVTLTALRALNLAHCMIDSLHSLIRCPLPAITALNLRAN
FG07231.1 	LSNTLYSLDISSNLFSQVPDSLATLTALRALNLSHCMIDSLQSLTRNPLPAITALNLRAN
AN0409.1  	VANTLHSLDLSTNLFTEVPDSLASLIALRALNLSNCMIESLHSLSRNPLPAITALNLRAN
          	::*:*:***:*:***:::****.:* *******::***:**:** * *************

MG10199.1 	RLQSIAAIEKLYPLERLDLRDNKLTDPMELARLTNIPDLREVYVEGNPFTRTHKDYRVTI
NCU00046.1	RLTSIVGIEKLYPLERLDLRDNQITDPMELARLTGIPELREIYVEGNPFTRIHRDYRITI
FG07231.1 	RLQSLAGVERLVPLERLDLRDNRLVDPMELARLTGIPDIHEIWVEGNPFTRTHKDYRITI
AN0409.1  	RLRSLAGIERLLSLERLDLRDNKLSDPMEIARLTSLPEIREIWVAGNPFVKTHPNYRVVI
          	** *:..:*:* .*********:: ****:****.:*:::*::* ****.: * :**:.*

MG10199.1 	FNLFRQTPGYTEDITIDSTGPTYAEKKQLVDRVPLPPAVPVVKPRASEIPAVDVLKPAII
NCU00046.1	FNLFRQTPGYTEDITIDGSGPSYSERRYLHERAAELPAVPVIKPPPQELPAVDVSKPTFV
FG07231.1 	FNLFRKAPGYTEDVVIDGSGPSYVERRSLVDRPPMPESIPVVKPLAPEVPTVDVSKPTVV
AN0409.1  	LNLFRRTPGYSEDIIIDGSGPGFTERKQLIERAAEPGVVPVIRSTVADN-STLVSKPSVT
          	:****::***:**: **.:** : *:: * :* .    :**::.   :  :. * **:. 

MG10199.1 	YETP--KEASVLRKERPRPKATASEINTNSTRRRRAPKRRIVDLATKESLAANGQPTEFT
NCU00046.1	YEPPP-REPAVLRKERPMPKSTTSELNTASTRRRKAPKRRIVDLATNETPAPVTLVDDRS
FG07231.1 	YNIP--KESTVLRKERPTPKAVMSEINTSSTRRRKTPKRRIVDLATPDAPPFPTPVDVQV
AN0409.1  	PASAASATRPAQDVDASRAEHLANDNGIGSSRRKRNNRKKIIDQSGAASIDGDRDSGAVV
          	   ..:   ..   : . .:   .: .  *:**::  :::*:* :   :           

MG10199.1 	SQLVKSSQGRVIYKEDNYRISQTADSQSPQLSAPVS---ITKVPATTQEVPRINTGILKT
NCU00046.1	RPGERAASG----HNEHYRISQPTEIHASQETLAAS---PLPDARAPADIPRIESTVIPS
FG07231.1 	AKLPNAALVN----DGNYRIFQQPDAQ-PLPAAVLS---PNSHIVTSPEVPRIDTNVFAN
AN0409.1  	PSVFSAQDPQ---LPVDPFVSSPTDSQWKSDGGPQAGSFHLQGPGAAKKKSRDDSAVPAS
          	     :          .  : . .: :        :.:       :. . .* :: :  .

MG10199.1 	DAAKQPVVVASHEPEPSWEASPPVDWDAGGEVYRRKIEELRDKVGQGYLSVLSEEGWDTS
NCU00046.1	ITPLYPSHKSSSESQ-SWRDNG--HWDVDGELYRRKIESLRDKVGSRYLSALNEEGWDPT
FG07231.1 	IPAIY----SPSDDAPNWTEPQ--DWDAGGEMYRQKIEALRSSVGNGYLSVLSEESWTPD
AN0409.1  	EFVLQ-------------QTLQSLEWGVDSDLQKHQLTALQQELGSSFFAALRDHAWNQA
          	                      . .*....:: ::::  *:..:*. :::.* :..*   

MG10199.1 	RHLQSHFQD-VQFGAPSPAMRQDPTAPR--VVQPIHAGRTLG-
NCU00046.1	-SLPPNYCE-GNFGAPSPLLA-DPTAPRAASVQAIHSGRTLG-
FG07231.1 	RSSDYSPPS-IPSGAVMHTAHPPIHTPQ---VQPIHSGRTLG-
AN0409.1  	QKTVAVPRTGTNFSESSRLSPESLTRAN---TQPILSGRHPIV
          	         .   .            ..   .*.* :**
```
